# High frequency in vitro regeneration of adventitious shoots in daylilies (*Hemerocallis sp*) stem tissue using thidiazuron

**DOI:** 10.1186/s12870-020-2243-7

**Published:** 2020-01-20

**Authors:** Kanyand Matand, Meordrick Shoemake, Chenxin Li

**Affiliations:** 1 Center for Biotechnology Research and Education, Langston, USA; 2 Undergraduate Student in the Department of Agriculture and Natural Resources, School of Agriculture and Applied Sciences, Langston University, Langston, OK 73050 USA; 3 Department of Plant Biology, College of Biological Sciences, University of California, Davis, CA 95616 USA

**Keywords:** In vitro plant regeneration, Daylilies tissue culture, Shoot organogenesis

## Abstract

**Background:**

Daylilies are a lucrative crop used for its floral beauty, medicinal proprieties, landscaping, fire prevention, nutritional value, and research. Despite the importance, daylilies remain extremely challenging for multiplying in vitro. The response difficulty is exacerbated because a few good protocols for daylilies micropropagation are generally difficult to reproduce across genotypes. An efficient strategy, currently applied at Langston University, is to systematically explore individual tissues or organs for their potential to micropropagation. This article is a partial report of the investigation carried out under room environmental conditions and focuses on developing an efficient daylilies in vitro propagation protocol that uses the stem tissue as the principal explant.

**Results:**

In less than three months, using thidiazuron, the use of the stem tissue as the in vitro experimental explant was successful in inducing multiple shoots several folds greater than current daylilies shoot organogenesis protocols. The study showed that tissue culture can be conducted successfully under unrestricted room environmental conditions as well as under the controlled environment of a growth chamber. It also showed that splitting lengthwise stem explants formed multiple shoots several folds greater than cross-sectioned and inverted explants. Shoot conversion rate was mostly independent of the number of shoots formed per explants. The overall response was explant and genotype-dependent. Efficient responses were observed in all thidiazuron treatments.

**Conclusion:**

An efficient protocol, which can be applied for mass multiple shoots formation using the daylilies stem tissue as the main explant, was successfully developed. This could lead to a broad and rapid propagation of the crop under an array of environmental conditions to meet the market demand and hasten exogenous gene transfer and breeding selection processes.

## Background

Daylilies (*Hemerocallis sp* L.) belong to the family Hemerocallidaceae and genus *Hemerocallis* [1]. It is a crop of economic importance used for natural beauty [2, 3], landscaping [4, 5], food nutrition [6–9], medicinal proprieties [10–12], and research.

Like many other crop species of economic value, daylilies have been explored for rapid and more efficient in vitro multiplication to better meet the market demand [13–16]. Although there have been successes, daylilies are conspicuously difficult to propagate in vitro using tissue culture approaches. In general, there are no peer-reviewed publications on daylilies micropropagation in the last five years or on stem tissue in the last seven years. However, overall, there have been two peer-reviewed publications on the daylilies stem in recent decades [15, 17], which makes this report unusually significant. The daylilies stem is the leafless flower stalk that grows directly from the crown. It is a temporary structure restricted to the reproductive cycle. This predictably makes studying in vitro totipotency limiting and challenging.

The challenges of daylilies micropropagation are exacerbated because a few good protocols that have been developed are generally difficult to reproduce across genotypes. This difficulty might be partly because the most valuable varieties are interspecific hybrids**.** Further, the difficulty of de novo plant regeneration in vitro might also explain the broad complaints by daylilies farmers and enthusiasts about the inconsistencies of true-to-type inflorescences and overall profiles of several plants purchased as in vitro tissue culture-produced [18–21].

**Fig. 1:**
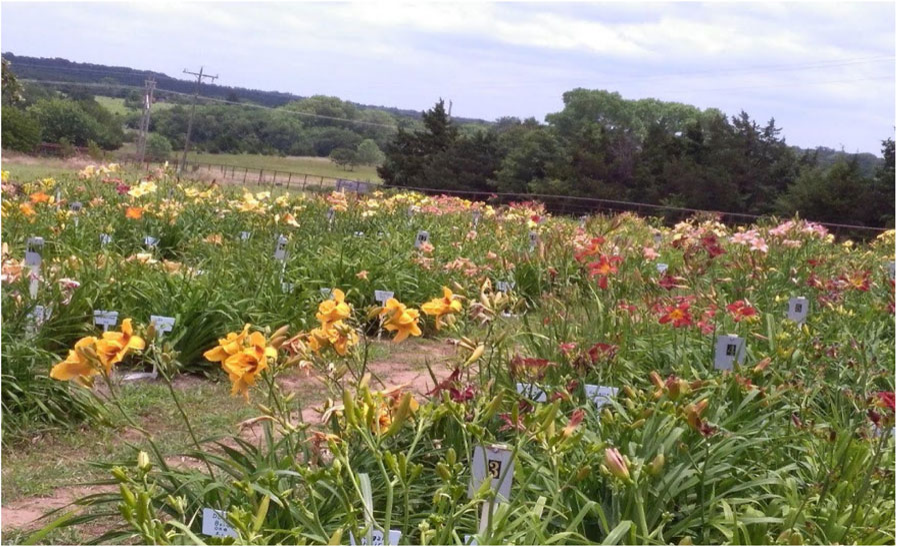
– Showing daylilies field genetic stock that provided lab fresh stem tissues

Langston University (Langston, Oklahoma, U.S.A.) recently established a large daylilies genetic stock of more than 250 varieties (Fig. 1) for a comprehensive screening for in vitro totipotency to develop a general protocol that can be applied across genotypes, irrespective of the geographic location and/or climatic conditions. Herein we report our progress on daylilies in vitro shoot organogenesis, applying the stem tissue as the experimental explant influenced by thidiazuron (TDZ). Thidiazuron was selected because of its potential to effect broad tissues and genotypes even at lower concentrations, as evidenced in different studies [22, 23].

## Results

**Fig. 2:**
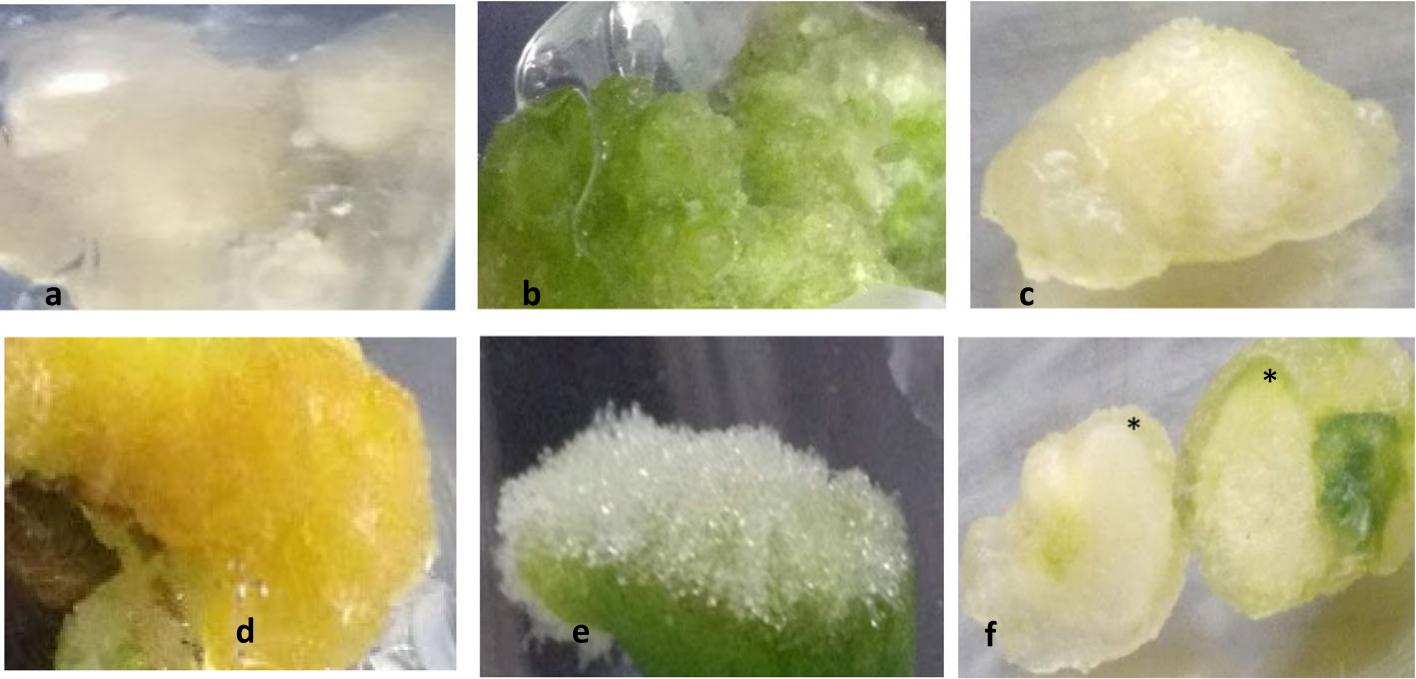
– Showing variable calli based on color and/or texture: **a** creamish, **b** green, **c** whitish, **d** yellowish and **e** snow; and **f** meristem developing through the greening outer layer of whitish callus (*shows developing meristem ring in the cross section of the whitish callus)

**Fig. 3:**
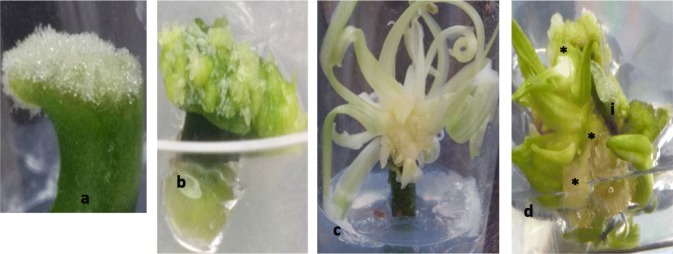
– Showing transitory and antecedent roles of snow callus in IC explants **a-c**; it disappeared as multiple shoots formed **b** and developed **c** and did not obviously makeup new shoots as did normal callus in CUEU explants **d**. *shows normal callus forming multiple shoots at the base of the cross-sectioned stem explant (i) cultured with the upper end up to keep stem normal polarity

**Fig. 4:**
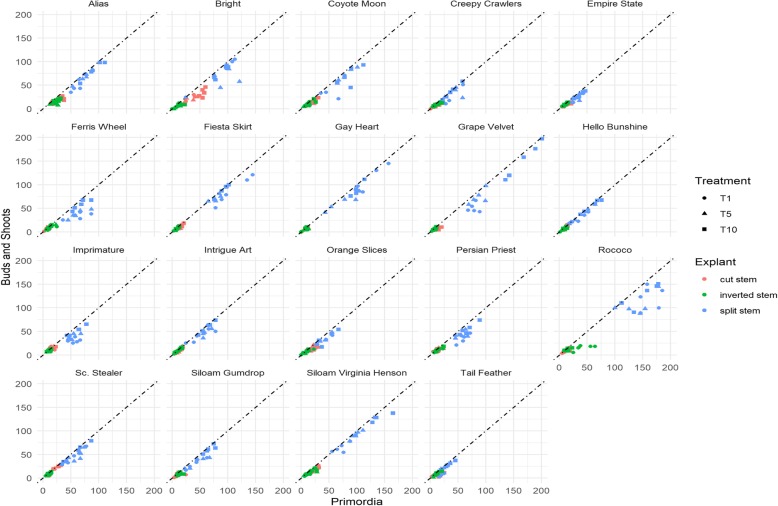
– Showing interactive responses of shoot primordia and shoot buds and shoots to thidiazuron (TDZ), variety and explant type: X axis = number of shoot primordia, Y axis = number of shoot buds and shoots; T1 = 1 mg/l TDZ, T5 = 5 mg/l TDZ, T10 = 10 mg/l TDZ; cut stem = cross-sectioned stem cultured with the upper end up (CUEU), inverted stem = cross-sectioned stem cultured in inverted position (IC), split stem = length-sectioned stem (LWS); mean differences were tested at 5% level of significance with Tukey Test using R and R Studio software

**Fig. 5:**
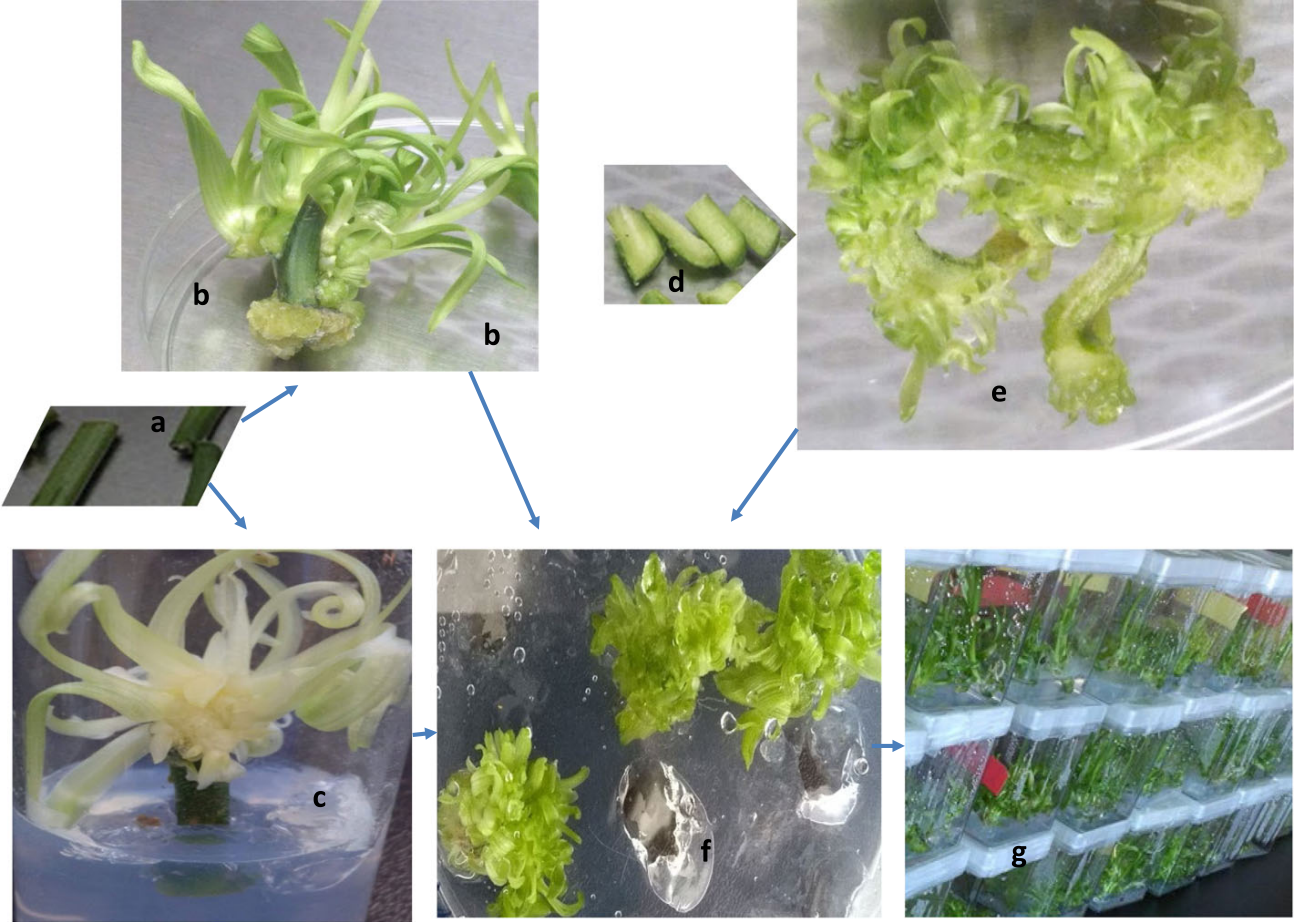
– Showing shoot formation pattern: **a** initial fresh tissue that was used as a cross-sectioned stem cultured with the upper end up (CUEU) or cross-sectioned stem cultured in inverted position (IC) explant to induce multiple shoots; **b** a CUEU explant with multiple shoot buds and shoots growing at the explant base inserted into the medium; **c** an IC explant with multiple shoot buds and shoots growing at the aerial base of inverted explant; **d** initial fresh tissue that was used as LWS explant, for culture; **e** a growing length-sectioned stem (LWS) explant with a more expanded, twisted wound surface area with multiple shoot buds and shoots along length-sectioned wound; **f** sample clusters of multiple shoot buds and shoots subdivided from a growing CUEU **b**, IC **c** or LWS **e** explant, for continuous induction of repeated multiple buds and shoots; **g** repeated multiple shoot buds and shoots resulting from subdivisions of multiple buds and shoots clusters in **f** of a single LWS explant

**Fig. 6:**
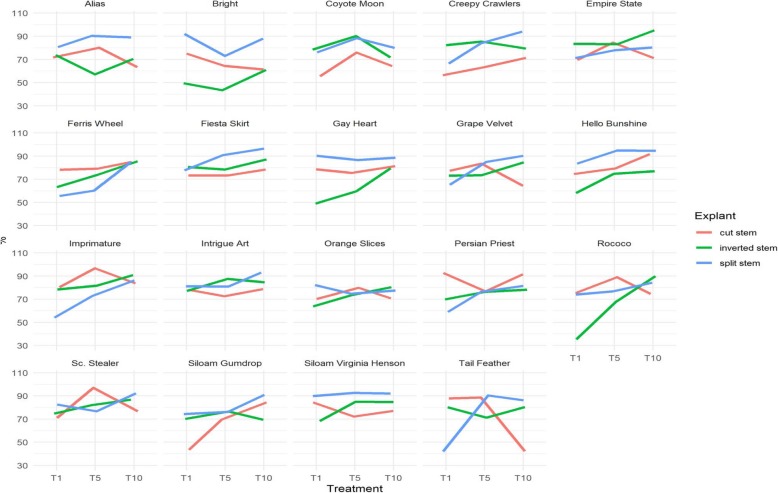
– Showing interactive responses of shoot buds and shoots conversion rates (%) to thidiazuron (TDZ) treatment (T1, T5, T10), variety and explant type (cut stem, inverted stem, split stem); T1 = 1 mg/l TDZ, T5 = 5 mg/l TDZ, T10 = 10 mg/l TDZ; cut stem = cross-sectioned stem cultured with the upper end up (CUEU), inverted stem = cross-sectioned stem cultured in inverted position (IC) and split stem = length-sectioned stem (LWS); mean differences were tested at 5% level of significance with Tukey Test using R and R Studio software

**Fig. 7:**
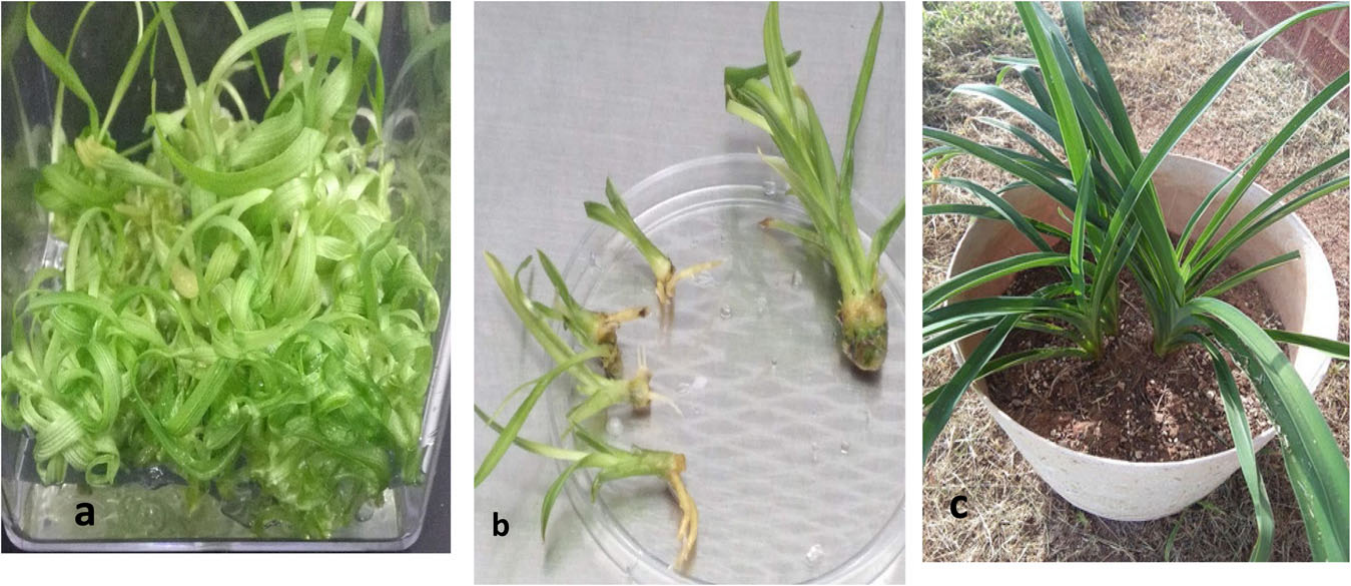
– Showing advanced growth of mass multiple plants: **a** several tens of shoot *buds* and *shoots* from a single subdivision shoots-cluster (see Fig. 5f) from a length-sectioned stem (LWS) explant, **b** rooted and non-rooted shoots, and (c) potted plants growing normally

The present data result from the interactions of three variables that include TDZ, genotype and explant, and are presented in Figs. 2 to 7. As a growth regulator, TDZ was used to initiate extracellular signals that were perceived and transduced into cell developmental structures, such as calli, organ primordia, buds and shoots, and roots that underscored core observations we made during the study. Following are salient observations.

### Callus formation and profile

The study showed that six varieties out of nineteen, including ‘Scene Stealer’, ‘Bright Banner’, ‘Grape Velvet’, ‘Orange Slices’, ‘Intricate Art’, and ‘Empire State’, formed significant calli; of those six, ‘Scene Stealer’ formed the most callus**.** Observed calli were categorized into five types that included creamish (Fig. 2a), green (Fig. 2b), whitish (Fig. 2c), yellowish (Fig. 2d), and snow (Fig. 2e). The first four categories of callus were permanent and mostly friable; thus, were described as normal. On the other hand, snow callus was ephemeral, not friable, and could not be multiplied independently. Therefore, it was described as abnormal. Further, normal calli were foundational to shoot formation. Shoots that formed in creamish and yellowish calli developed the green color progressively with growth. Snow callus was precursory to abundant multiple shoot formation in both length and cross-sectioned explants**.** Unlike other calli, whitish callus developed a greening meristem ring or outer layer that was distinct in the cross-section (Fig. 2f) and preceded prolific shoot organogenesis.

### Shoot organogenesis

Shoot organogenesis was the primary path for plant formation, as somatic embryogenesis was not observed. Although there were shoots that formed via callus, prominent shoot formation occurred directly from differentiated tissues. Considering the transitory and antecedent nature of snow callus, related shoots were also categorized as direct (Fig. 3a-c).

### Explant cultural period to shoot organogenesis

Overall, shoot organogenesis was observed in genotypes within one to five weeks of culture. Varieties including ‘Orange Slices’, ‘Imprimatur’, ‘Bright Banner’ and ‘Fiesta Skirt’, ‘Ferris Wheel’ and ‘Empire State’, and ‘Alias Peter Parker’, ‘Gay Hearted’ and ‘Persia Priest’ formed shoot primordia within 8, 10, 11, 12, and 13 days, respectively. The culture period for observing shoot organogenesis in the rest of the varieties ranged from 14 to 35 days. Although the initial observation of shoot organogenesis occurred within a week of culture, more than half of the varieties studied induced organ formation within two weeks.

#### Shoot primordia, shoot buds and shoots

The formation of shoot primordia is quintessential for subsequent shoot buds and shoots development. The number of shoot primordia that cumulatively formed throughout the study period ranged from 0 to 201 per explant. The variety ‘Grape Velvet’ induced the greatest explant number of shoot primordia. The top five varieties with greatest averages per explant included ‘Grape Velvet’ (167), ‘Rococo’ (167), ‘Siloam Virginia Henson’ (131.8), ‘Gay Hearted’ (120.6), and ‘Fiesta Skirt’ (108.8). The development of shoot buds and shoots are the most coveted steps in the shoot organogenesis process because it physically manifests plant formation and requires only one additional step, root formation, to complete the whole plant formation process. The average shoot buds and shoots per explant ranged from 3 to 152.2 (Fig. 4). The top five varieties with greatest explant averages of shoot buds and shoots included ‘Grape Velvet’ (152.2), ‘Rococo’ (127.5), ‘Siloam Virginia Henson’ (120.8), ‘Gay Hearted’ (109.2), and ‘Fiesta Skirt’ (89.4). The response per individual explants is generally a reliable indicator of the level of success of a study. Although significant, this variable is often overlooked in plant micropropagation studies. Such an omission generally makes it very challenging to assess the efficacy of the protocol that was applied in a given study.

#### Effect of explant conditioning

##### On shoot organogenesis

The stem was successfully conditioned as CUEU (Fig. 5a→b), IC (Fig. 5a → c), and LWS (Fig. 5d→e) explants for shoot organogenesis (see also Methods). Like the genotype and treatment, explant conditioning had a significant effect on shoot buds and shoots formation (Fig. 4). LWS explants induced more shoots compared with CUEU and IC. On average, the number of buds and shoots that formed in LWS explants ranged from 8 to 152.2, whereas those that formed in CUEU and IC were 4.8–34.2 and 4.2–17.0, respectively (Fig. 4). We also observed that splitting the stem explant lengthwise yielded the greatest total organogenic response that was five and six folds greater than CUEU and IC, respectively. Of the three explant types, LWS had the broadest wound that expanded several folds throughout the culture (Fig. 5e). Mitosis in wound cells occurred exponentially (in attempts to heal it) compared to the cells on the backside within the uncut epidermis of the same explant unit. The disproportionate cellular growth generated greater tension within the epidermis that compelled growing individual LWS explants (Fig. 5e) to bulge out, which resulted in longer twisted explants with more expanded wound surface area that was amenable to greater shoot organogenesis compared with CUEU (Fig. 5b) or IC (Fig. 5c). Individually responding explants were frequently subdivided into smaller subunits that resulted in forming repeated multiple shoot buds and shoots clusters (Fig. 5f and g).

##### On callus and shoot formation pattern

There was a correlation between explant conditioning and shoot formation pattern. Shoot primordia and shoots formed directly along length-sectioned wounds of the LWS explants (Fig. 5d and e). Those explants formed the greatest numbers of shoot primordia and shoots, irrespective of the treatment and genotype (Fig. 4). Snow callus was prominent and precursory to multiple shoots in the length-sectioned wound of LWS or cross-sectioned wound of IC explants. Normal callus formation was not apparent in IC explants. Occasionally the IC’s end that was inserted into the medium expanded into dividing mass cells that were described as non-callus (Fig. 3b), because they were not friable and could not independently survive in the culture medium when they were excised and sub-cultured from the original explant. This supports our hypothesis that the inverting of the stem explant might have interfered with the formation of normal callus. However, the same disruption of the stem polarity did not affect the potential to form shoots. Further, multiple shoots formed directly or indirectly at the CUEU explant’s base that was inserted into the culture medium (Fig. 3 and 5d and b). Similarly, IC explants formed shoots at their base, which in this case was the aerial end of the explant (Figs. 3 and 5c and c).

#### Effect of thidiazuron content level

Three TDZ concentrations studied included 1, 5, and 10 mg per liter of culture medium. The study showed that TDZ was effective in forming shoots in daylilies stem tissue, irrespective of the concentration, genotype, or explant conditioning (Fig. 4). It induced broad shoot primordia and shoots that were genotypically dependent. The lowest concentration, 1 mg/l TDZ, formed shoot primordia of averages ranging from 6 to 167 (Fig. 4) per explant, including the greatest average (167). The averages of corresponding shoot buds and shoots ranged from 3 to 127.5 per explant (Fig. 4). The 10 mg/l TDZ concentration induced the greatest number of shoot primordia (8.2–167) and shoot buds and shoots (7–152.2) per explant. It also caused the second greatest shoot conversion rate (43.75–95.70%) (Fig. 6) and the shortest period to shoot organogenesis (8 days). Like 1 and 10 mg/l, the 5 mg/l TDZ concentration induced great shoot organogenic responses. The averages of its shoot primordia ranged from 6.8 to 140.4 per explants and those of shoot buds and shoots per explant ranged from 4.4 to 105.8 (Fig. 4). However, it also caused the longest period (35 days) to shoot organogenesis. During the study, we occasionally observed signs of hyperhydricity in explants across TDZ concentrations. However, it was temporary and easily controlled by shortening the subculture cycle. It also disappeared with shoot root formation.

#### Shoot buds and shoots conversion from shoot primordia

To determine the efficacy of explant conditioning and the potential of individual varieties to induce shoot organogenesis, we calculated the conversion rates by dividing the number of shoot buds and shoots by the number of shoot primordia and multiplying the quotient by 100. Data are presented in Fig. 6. On this standpoint, ‘Scene Stealer’ had the greatest shoot conversion rate (96.08%) followed by ‘Creepy Crawlers’ (95.70%), ‘Empire State’ (94.64%), ‘Imprimature’ (94.52%), and ‘Hello Bunshine’ (94.16%) (Fig. 6). The results further showed that shoot buds and shoots conversion potential was genotypically dependent, and the best shoot inducers were not necessarily the best shoot converters. These results may explain why none of the top five varieties with greatest shoot primordia or shoot buds and shoots were among the top five shoot buds and shoots converters. The study also explored whether there was a correlation between shoot conversion potential and explant conditioning. Numerically, CUEU explants converted shoot primordia into buds and shoots at the greatest rates that ranged from 42.11 to 96.08%, which were followed by LWS and IC explants with 42.55–95.70% and 35.02–94.64%, respectively. However, the conversion rates were not statistically different between CUEU and LWS. Overall shoot conversion potential appeared to be independent of the TDZ concentration, considering that 1, 5, and 10 mg/l TDZ were responsible for 42.11–90.79%, 51.16–96.08%, and 43.75–95.70% conversion rates, respectively (Fig. 6).

#### Root formation

This study overall resulted in a scant root formation, considering that only four varieties (‘Alias Peter Parker’, 17.00%; ‘Coyote Moon’, 39.13%; ‘Imprimatur’, 29.40%; and ‘Rococo’, 48.17%) had a limited number of root-forming shoots during the experimental period. Occasionally, roots were observed forming directly from CUEU explants without shoot organogenesis. All plantlets that formed roots grew normally (Fig. 7).

## Discussion

The present study investigated whether daylilies stem tissue can be used reliably as an in vitro explant for micropropagation. Our report shows encouraging broad and great shoot organogenic responses that support the hypothesis. The success of the study should particularly underscore the potential of an unrestricted lab, non-controlled environment in which the study was carried out. This could level with routine practices requiring controlled environmental conditions of the growth chamber for success, and therefore, encourage greater in vitro plant tissue culture studies even under the most financially prohibitive conditions of developing countries.

Thidiazuron was used as the principal exogenous source of chemical signals that were transduced into cellular morphologies we observed. It is a phenyl-urea compound that has been substantiated to have the cytokinin potency across plant species [24–27]. Growth regulators’ signal conversions into metabolic activities and developmental morphologies in cells or organisms have been established in biology [28–33]. The concept is broadly utilized for downstream applications particularly in cell, tissue, and organ cultures as well as plant transformation across plant species [24, 34–37]. Generally, TDZ is used at lower concentrations to effect plant micropropagation [23, 26, 27, 38]. The concentrations smaller than 1 nM have proven to be effective in inducing shoot organogenesis [39]. However, it is evident in these studies that even at a concentration several folds greater than frequently applied [40–42], TDZ produced even greater results. The results suggest that there might be an upper limit of TDZ concentration that has not yet been determined in daylilies micropropagation. Therefore, more studies are encouraged to determine the related threshold concentration. Furthermore, all TDZ concentrations that were studied yielded equally great results such that any of it is recommended for success.

The application of TDZ was primarily intended for shoot organogenesis. Although most shoots formed directly, variable organogenic calli developed during the studies. This is not unusual in plant tissue culture, considering that several protocols for shoot organogenesis in plant species involve an intervening callus phase [43–46]. Generally, callus formation is an inherent wound healing mechanism that has been evidenced in plants [47–49]. Although it generally forms endogenously without subsequent organ formation [50], there is evidence of some plant non-hormonal callus conducing to organ regeneration [51]. In the present study, callus formation was caused by TDZ. The color and texture were used to categorize calli and predict its organogenic potential. All normal calli promoted shoot organogenesis. Considering that there was no significant normal callus that formed in inverted explants, it suggested that the disruption of the stem normal polarity or orientation might have also disrupted normal callus formation.

Higher organisms have the capacity to replace damaged cells, tissues, or whole organisms through regeneration mechanisms [49, 52–54]. In plants, this mechanism is generally associated with wound stress and was noticeable in the studies considering that most shoot organogenesis occurred in wound areas. Accordingly, length-sectioned tissues were statistically the most effective and efficient explant type, because they induced and developed maximal numbers of primordia and shoots across explant types studied. Although the application of daylilies stem explant for de novo shoot organogenesis is generally wanting, there are two investigations of daylilies stem tissue culture that have been reported [15, 17]. In the studies by Lin et al. [17], the author observed about 6 shoots forming per explant. However, those shoots were the result of the sprouting of pre-existing axillary buds. The studies also mentioned the formation of callus from pre-existing meristem with subsequent shoot formation; however, no related specific count of shoots was reported. In the other studies by Chen et al. [15], meristematic clusters that formed in daylilies’ pedicel explants under the influence of a growth retardant developed an average of 28 shoots per meristematic cluster. However, the number or rate of meristematic clusters that formed shoots was not provided. In the present study, all genotypes formed shoot primordia with subsequent development of multiple buds and shoots. In addition to shoot organogenesis [55–61], the study showed that the explant response period was also genotype dependent. The variety that induced most primordia and multiple shoots during the studies was ‘Grape Velvet’. Regardless of the variations, cumulative shoot organogenic responses to individual TDZ concentrations were significant in the general context of plant tissue culture and more effective than previous studies on daylilies stem tissue [15, 17]. Therefore, either concentration is recommended for comparable studies or more efficient results.

Finally, because the studies did not include data beyond the experimental cut off (69 days), the scarcity of root formation we recorded could be leveled by abundant root formation that was observed during the post-experimental period.

## Conclusions

This is a partial report of daylilies broad cell, tissue and organ culture studies that are carried out at Langston University (Langston, OK, U.S.A.). It is the broadest successful investigation of the daylilies stem tissue in vitro and has confirmed its reliability as an in vitro explant for abundant multiple shoot formation. However, considering that daylilies stem is transitorily present on the crop during the growing season, using it in micropropagation should be linked to the crop’s reproductive cycle, which depends upon the geographic location and climatic conditions. Furthermore, the studies showed that even under unconstrained room environmental conditions, daylilies stem tissue can form more effective shoot organogenesis. Shoot organogenesis, shoot induction period, and TDZ concentration were genotypically dependent. Finally, ongoing research is necessary to clarify inconsistencies and enhance the understanding of the micropropagation of daylilies.

## Methods

### Plant materials

Experimental plant materials were daylilies genotypes collected from the local Langston University plant genetic stock. Nineteen varieties that were studied included ‘Alias Peter Parker’, ‘Sloam Gumdrop’, ‘Imprimatur’, ‘Persian Priest’, ‘Fiesta Skirt’, ‘Coyote Moon’, ‘Orange Slices’, ‘Siloam Virginia Henson’, ‘Creepy Crawlers’, ‘Tail Feather’, ‘Empire State’, ‘Intricate Art’, ‘Hello Bunshine’, ‘Ferris Wheel’, ‘Bright Banner’, ‘Scene Stealer’, ‘Gay Hearted’, ‘Grape Velvet’, and ‘Rococo’.

### Explant preparation and cultural conditions

The stem tissue was applied in this study as the experimental explant. The plant tissues were freshly collected, washed under running tap water until dirt and debris were fully cleaned off, and surface-sterilized with 35% sodium hypochlorite bleach (commercial Clorox) for 8–10 min; the materials were rinsed three to four times with sterile distilled water. Sterile stems were cross- and length-sectioned into 0.5–10 mm long explants under sterile conditions of a laminar air-flow hood (Baker Company, Inc.). Explants were cultured in Petri dishes or Magenta 7 (G7) containers of nutrients medium consisting of Murashige and Skoog (MS) salts and vitamins [62], sucrose (20 g/l), and thidiazuron (TDZ) wrapped with parafilm. Individual dishes/G7 contained ten explants; five dishes / G7 were randomly assigned to individual treatments. During the culture, when signs of contaminations were observed, explants of the suspected containers (dishes / G7) were washed with 5% Clorox and sub-cultured into independent test tubes with the same medium to control the rescue process and contain the spread of contaminations. However, related data were counted for the original containers they were rescued from. Explants were partitioned into three types based on the preparation and/or positioning onto the culture medium, which included cross-sectioned stem cultured with the upper end up (CUEU), cross-sectioned stem cultured in inverted position (IC), and length-sectioned stem (LWS). Each explant was used as an experimental unit for observations and data collection. The final pH of the medium was adjusted to 5.9 with 1 M NaOH after the addition of phytagel (4 g/l); the media were autoclaved at 121 °C for 20 min. MS medium was supplemented with 0, 1, 5, or 10 mg/l TDZ. Cultures were incubated at room environmental conditions (temperature, light, humidity, etc.). Explants were sub-cultured onto fresh media every four to six weeks. All chemicals were purchased from Sigma Co. (St. Louis, MO).

### Data observations and collections

Experimental observations and data collections were made daily on individual dishes/G7/test tubes for 69 days of experimental cut. Data reported were cumulated throughout the study. No data collected beyond the experimental period were included in this report. During the experiment, we used a light microscope (Stereomaster, Fisher Scientific) for counting shoot primordia and shoot buds and shoots. Because of the lack of related camera, we used a standard AT&T GoPhone photo camera to take pictures. Consequently, shoot primordia and most early shoot buds do not appear in the publication pictures due to the limited magnification capacity. However, they were accounted for in the data we are reporting.

### Experimental design and statistical analysis

Data from three parallel experiments, conducted with the same materials such as genotype, explant tissue, cultural medium, and growth regulator treatments under similar cultural and environmental conditions (room environment), were combined for this report. Further, data from individual explants were pooled into an average per culture medium container; the average of individual containers was used as a replicate value during the analysis. Therefore, five replicates per treatment were used for analysis. Our experiments were carried out using the randomized factorial design. Data were analyzed using generalized linear models with log link with interactions among factors. The analyses were performed with R and R Studio software (version 3.6.0; 2019-04-26). The statistical package used for analysis of variance was *emmeans* [63] and that for plotting and graphs was *ggplot2* [64]. The significance of mean differences was tested using the Tukey Test at 5% level.

## Data Availability

The datasets used and/or analyzed during the current study are available from the corresponding author on reasonable request.
